# Vis/NIR hyperspectral imaging distinguishes sub-population, production environment, and physicochemical grain properties in rice

**DOI:** 10.1038/s41598-020-65999-7

**Published:** 2020-06-09

**Authors:** Jinyoung Y. Barnaby, Trevis D. Huggins, Hoonsoo Lee, Anna M. McClung, Shannon R. M. Pinson, Mirae Oh, Gary R. Bauchan, Lee Tarpley, Kangjin Lee, Moon S. Kim, Jeremy D. Edwards

**Affiliations:** 1 Dale Bumpers National Rice Research Center, United States Department of Agriculture - Agricultural Research Service, Stuttgart, AR 72160 USA; 2 Environmental Microbial and Food Safety Laboratory, United States Department of Agriculture - Agricultural Research Service, Beltsville, MD 20705 USA; 3 Department of Biosystems Engineering, Chungbuk National University, Cheongju, 28644 Republic of Korea; 4 Electron & Confocal Microscopy Unit, United States Department of Agriculture - Agricultural Research Service, Beltsville, MD 20705 USA; 5 Grassland and Forages Division, National Institute of Animal Science, Rural Development Administration, Cheonan, 31000 Republic of Korea; 6 Texas A&M AgriLife Research Center, Texas A&M University System, Beaumont, TX 77713 USA; 7 National Institute of Horticultural and Herbal Sciences, Rural Development Administration, Haman, 52054 Republic of Korea

**Keywords:** High-throughput screening, Genome-wide association studies, Plant breeding

## Abstract

Rice grain quality is a multifaceted quantitative trait that impacts crop value and is influenced by multiple genetic and environmental factors. Chemical, physical, and visual analyses are the standard methods for measuring grain quality. In this study, we evaluated high-throughput hyperspectral imaging for quantification of rice grain quality and classification of grain samples by genetic sub-population and production environment. Whole grain rice samples from the USDA mini-core collection grown in multiple locations were evaluated using hyperspectral imaging and compared with results from standard phenotyping. Loci associated with hyperspectral values were mapped in the mini-core with 3.2 million SNPs in a genome-wide association study (GWAS). Our results show that visible and near infra-red (Vis/NIR) spectroscopy can classify rice according to sub-population and production environment based on differences in physicochemical grain properties. The 702–900 nm range of the NIR spectrum was associated with the chalky grain trait. GWAS revealed that grain chalk and hyperspectral variation share genomic regions containing several plausible candidate genes for grain chalkiness. Hyperspectral quantification of grain chalk was validated using a segregating bi-parental mapping population. These results indicate that Vis/NIR can be used for non-destructive high throughput phenotyping of grain chalk and potentially other grain quality properties.

## Introduction

Rice grain quality influences crop value and is important to growers, millers, and processors as well as consumers^1^. Grain quality in rice is determined by multiple factors including starch composition, cooking quality, and grain size, shape, and translucency (chalky appearance)^2^. High grain chalk causes grain breakage during milling and loss of crop value impacting domestic and export markets^3^. Molecular markers are sought as tools for marker-assisted selection (MAS) in rice breeding for traits like grain quality that are complex, difficult to phenotype and are influenced by the production environment^4^.

Rice has a well-defined population structure with two distinct sub-species that are further divided into sub-populations^5^. The *indica* (IND) and *aus* (AUS) sub-populations belong to the sub-species Indica (AUS-IND) and the *tropical japonica* (TRJ), *temperate japonica* (TEJ), and *aromatic* (ARO) sub-populations belong to the Japonica sub-species (TEJ-TRJ). Even though there is wide variation within each of these categories, rice sub-species and sub-populations have phenotypic and genetic differences that influence their adaptation to different environments and are associated with physicochemical traits^6^. Having knowledge of sub-species and sub-populations in rice is important for breeding programs targeting different production environments and grain market (i.e. short, medium, and long grain markets) classes.

Genome-wide association mapping studies (GWAS) have been used in rice to map a wide range of traits^7,8^. Several rice diversity panels exist that are genotyped at a high density and are suitable for GWAS such as the 3000 rice genomes^9^, the High Density Rice Array (HDRA)^10^, and the USDA rice mini-core collection^8,11,12^. The USDA rice mini-core germplasm collection of 217 accessions is selected to be phenotypically and genotypically representative of the USDA worldwide rice collection^11,13^, and includes the five sub-populations of *O. sativa* (AUS, IND, TRJ, TEJ, and ARO). It has been re-sequenced to an average depth of 1.5×^8^ and has a filtered genomic dataset of 3.2 million single nucleotide polymorphic (SNP) markers^8,12^. Therefore, it is an excellent genetic resource to identify chromosomal regions associated with various phenotypic traits. However, analysis of diversity panels can have confounding factors that may mask or produce false associations with the phenotype of interest. Therefore, quantitative trait loci (QTL) mapping using bi-parental recombinant inbred line populations is a means to validate findings from GWAS studies.

One of the bottlenecks in mapping of genes for grain quality traits is the intensive labor, time, and expense required to phenotype the diversity of physicochemical traits impacting rice quality. Near-infrared (NIR) spectroscopy has been widely used to determine protein, amylose, oil, and moisture contents in rice whole grain and flour^14–16^ as well as other quality traits (i.e. sensory and starch pasting properties)^17^ and in other crops^18,19^. However, Vis/NIR spectroscopy is a rapid analytical tool that assesses samples by utilizing visible and near-infrared regions of the spectrum and has been demonstrated to be useful in detecting biotic and abiotic stress factors in plant products^20,21^.

The aims of this study were to (1) determine if Vis/NIR hyperspectral imaging of whole grain rice can accurately classify samples according to sub-population or production environment, (2) to determine if variation in Vis/NIR wavelengths correlates with grain quality traits, including chalkiness, and (3) to identify specific genomic regions that are associated with the variation measured through Vis/NIR that can be used to identify corresponding candidate genes.

## Materials and Methods

### Grain production

Grain samples used for hyperspectral imaging came from the USDA rice mini-core collection (221 accessions) including 38 AUS, 86 IND, 33 TEJ, 40 TRJ, 6 ARO, and 18 admixed accessions. The samples were grown in three environments, the USDA-ARS Rice Research Unit/Texas A&M Agrilife Research Center located in Beaumont, Texas in 2008 (TX08) and in 2009 (AR09) and 2010 (AR10) at the USDA-ARS Dale Bumpers National Rice Research Center located in Stuttgart, Arkansas. The cultural management practices for the TX08 study (League clay soil, fine, smectitic, hypothermic Oxyaquic Dystrudert) have been described by Pinson *et al*.^22^. Li *et al*.^23^ described the cultural practices used for the AR09 study which were the same for AR10 (unpublished) (Dewitt silt loam soil, fine, smectitic, thermic, Typic Albaqualf). Field plots were drill seeded to a depth of approximately 2 cm in hill plots in TX08 and in row plots in AR09 and AR10. At both locations, plots were irrigated after seeding to enhance uniform germination. When seedlings reached a height of approximately 9 cm, a permanent flood was applied for the remainder of the season. At the TX08 location, the total fertilizer applied was 73 kg ha^−1^ N as urea and 33.6 kg ha^−1^ P; whereas the fertilizers applied in AR09 were 55 kg ha^−1^ of N, 34 P kg ha^−1^, 67 K kg ha^−1^ and 11 Zn kg ha^−1^ ; and in AR10 were 55 kg ha^−1^ of N, 56 P kg ha^−1^, and 67 K kg ha^−1^ according to soil analyses and local recommendations. As plots reached maturity they were harvested by hand, threshed and dried to approximately 12% moisture prior to storage at 4 °C and 50% humidity as rough rice. Prior to imaging, grain samples stored at 4 °C were transferred to a desiccator for 2–3 days to prevent accumulation of moisture on the grain as it equilibrated to room temperature (10 °C) prior to imaging. Arkansas weather data in Stuttgart was provided from the United States Department of Agriculture (USDA) Agricultural Research Service Dale Bumpers National Rice Research Center (https://www.ars.usda.gov/southeast-area/stuttgart-ar/dale-bumpers-national-rice-research-center/docs/weather-data-archives/), and Texas weather information in Beaumont was from the National Weather Service (NWS) Cooperative Observer Program (COOP) (http://www.ncdc.noaa.gov/data-access/land-based-station-data/land-based-datasets/cooperative-observer-network-coop).

In addition, samples came from a field study using the bi-parental long grain mapping population KBNT lpa1–1 x Zhe733 recombinant inbred lines (KZ-RILs) that was conducted at the USDA-ARS Dale Bumpers National Rice Research Center/University of Arkansas Rice Research and Extension Center, Stuttgart, Arkansas^24^. The population of 187 KZ-RILs was planted on two planting dates, about 30 days apart, during 2013 and 2014 using a randomized complete block design with three replications. Details of the study are described by Edwards *et al*. (2017) and were similar to the AR09 and AR10 studies in terms of crop management, harvest methods, and grain analysis. For the current study, only samples from the second planting date in 2013 and the first planting date in 2014, were used. These two environments were determined to be the most diverse in terms of climatic differences and one field replication was used from each year. All rough rice samples were dehulled (Satake Rice Machine, Satake Engineering Co., Ltd, Tokyo, Japan) to produce at least 100 grains of brown rice for hyperspectral imaging. The seeds used for hyperspectral imaging came from the same seed source as those used for percent chalk but were different sub-samples.

### Hyperspectral imaging system and image acquisition

The reflectance values of brown rice samples were obtained using a visible and near infra-red (Vis/NIR) hyperspectral imaging system that was developed at the Environmental Microbiological and Food Safety Laboratory, Agricultural Research Service, USDA in Beltsville, MD, USA^25^. It consists of an electron-multiplying charge-coupled-device (EMCCD) camera (Luca, Andor Technology Inc. CT, USA), a spectrograph, six 100-watt halogen bulbs at a distance of 50 cm to the rice samples with 15° angle, and a translational stage^25,26^. The system acquires spectral wavelengths in the range of 400 nm to 1004 nm. Grain samples were dried under vacuum desiccator for 2–3 days prior to imaging to avoid any inconsistent moisture content across the samples. Ten samples (about 100 grains per sample) were taken per image. The exposure time for each sample was set to 5 milliseconds. The final size of the hyperspectral image was 502 × 600 pixels. The original hyperspectral image was calibrated for white and dark balance using the following equation:

1 $${\rm{I}}=\,\frac{{{\rm{I}}}_{0}-{\rm{D}}}{{\rm{W}}-{\rm{D}}}$$

where I is the relative reflectance of the hyperspectral image (scaled from no reflectance at a value of 0 to 100% reflectance at a value of 1), I_0_ is the original image data, W is the white-reflectance image data, and D is the dark-current image data. The region of interest (ROI) for the hyperspectral images was manually extracted at 750 nm, as this wavelength had the highest intensity with intact brown rice kernel. The binary image without background noise from non-grain areas of the image was used for obtaining the spectral information corresponding to each pixel in the hyperspectral data. Eight mathematical pre-processing methods; smoothing, mean normalization, maximum normalization, range normalization, multiplicative scatter correction, standard normal variate (SNV), Savitzky-Golay first derivative and Savitzky-Golay second derivative; were applied to the raw data before developing the optimal model^27–30^ Image correction, segmentation, and spectral-data extraction were customized using the MATLAB software^31^.

### Grain quality traits

Data on the physicochemical traits for the USDA rice mini-core collection used in this study were previously described in Huggins *et al*. 2019. This included apparent amylose content (AAC), ASV (an indicator of gelatinization temperature), grain length, width and thickness (mm) (N=197). Brown (unpolished) rice was used to determine percent chalk, and only non-pigmented bran (i.e. white, light brown, and brown) accessions of the mini-core (N= 137) were used because purple and red pericarp can mask the chalk phenotype. For the mini-core, an image analysis system as described in Edwards *et al*. (2017) was used to determine grain length and grain width on brown rice, whereas hand calipers were used to determine grain thickness on 20 kernels. Milled rice was used to determine AAC and ASV values. Grain quality trait data for the bi-parental mapping population KBNT lpa1–1 x Zhe733 have been previously described by Edwards *et al*. (2017) and include percent chalk measured using brown rice.

### Scanning electron microscopy analysis of grain chalk

Scanning electron microscopy (SEM) was used to validate the percent chalk as determined by the image analysis phenotyping method (described above) and determine if chalk differences in rice grains are associated with spectral differences. Brown rice samples from KZ-RILs (N=4) with diverse chalk phenotypes were analyzed as these did not differ dramatically for grain dimension or bran color like the mini-core accessions. Whole grains were cut longitudinally with a razor blade and placed on 15 × 30 mm copper plates using ultra smooth, round (12 mm diameter) carbon adhesive tabs (Electron Microscopy Sciences, Inc., Hatfield, PA, USA). The samples were transferred to the Quorum PP2000 cryo-prep-chamber (Quorum Technologies, East Sussex, UK) attached to an S-4700 field emission scanning electron microscope (Hitachi High Technologies America, Inc., Dallas, TX, USA). The specimens were coated with a 10 nm layer of platinum using a magnetron sputter head equipped with a platinum target in the Quorum PP2000. After coating, the specimens were transferred to the SEM for observation. An accelerating voltage of 5 kV with a working distance of 10 mm was used to view specimens. Images were captured using a 4pi Analysis System (Durham, NC, USA).

### Genome-wide association analysis

Whole-genome SNP data for the USDA mini-core diversity panel was obtained from resequencing by Wang *et al*., (2016). The raw reads generated by Wang *et al*., (2016) for 203 mini-core accessions were downloaded from the sequence read archive and called against the Michigan State University version 7 (MSU7) rice pseudomolecules^32^ using the Genome Analysis Toolkit (GATK)^33^ as described in Huggins *et al*., (2019). A set of approximately 3.2 million SNPs were generated after filtering loci with a missing rate over 20% and minor allele frequency (MAF) less than 0.05. Principal components (PC) and a kinship matrix were generated using the 3.2 million SNPs in TASSEL version 5^34^. The first three PCs and the kinship matrix were incorporated into a mixed linear model (MLM) to account for relatedness and population stratification. The GWAS analysis of the first principal component for spectral values in the range 702–922 nm spectral range was performed with an MLM that included the options “each marker” and “no-compression” to determine trait marker associations in TASSEL version 5^34,35^. Only the non-pigmented bran subset (N=132) was used for GWAS. The GWAS significance threshold for p-value was determined through false discovery rate (FDR) analysis using the R package ‘qvalue’^36,37^. Based on FDR analysis, the p-value threshold for genome-wide significance was set at 10^−6^ and only SNPs that met or exceeded this value were considered as significant. The p-values of the GWAS was visualized as Manhattan and Q-Q plots using the R package ‘qqman’^38^ (Fig. 6; Supplementary Fig. S4).

**Figure 1:**
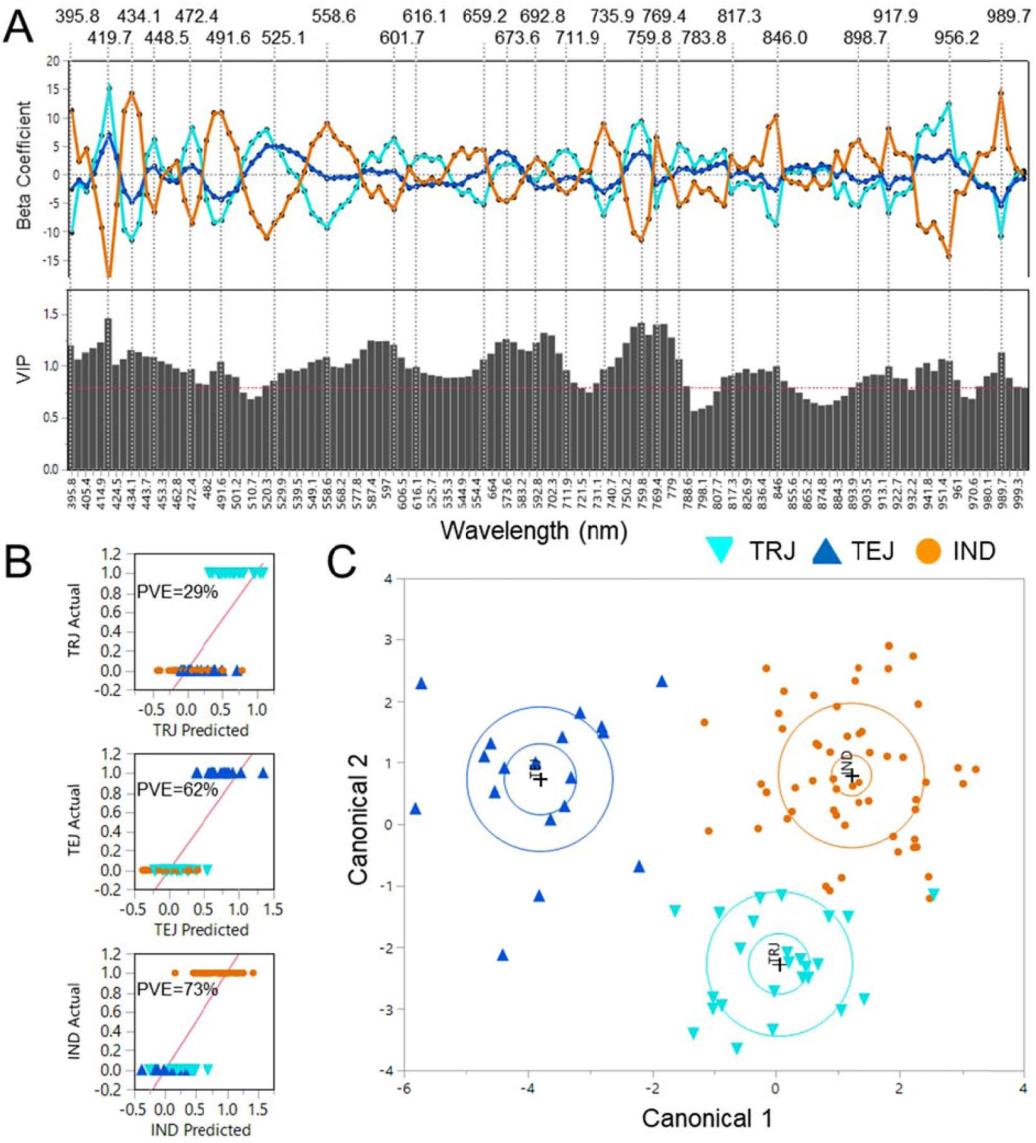
Regression beta coefficient and variable importance plot (VIP) of PLS-DA models that discriminate TRJ (cyan), TEJ (blue), and IND (orange) using centered and scaled Vis/NIR spectra with 24 selected predictive wavelengths indicated **(A)**. Population classification of TRJ, TEJ, and IND using PLS-DA models and percent variance explained (PVE) for each population **(B)**. Canonical plot for linear discriminant analysis of TRJ, TEJ, and IND using the 24 predictive wavelengths selected from the PLS-DA model **(C)**.

### Candidate genomic regions

The GWAS output was used to determine chromosomal regions that are significantly associated with grain quality traits. Chromosomal regions (loci) containing putative QTLs were defined as spanning from 100 kb on either side of a significant SNP and extended if additional significant SNPs were contained in the 100 kb and calculated with a Perl script as described in Huggins *et al*. (2019). The chromosome, start and stop positions, the most significant SNP (peak SNP) and the p-value of the region were output to a text file for each hyperspectral trait. Text files of identified chromosomal segments for hyperspectral traits and grain quality traits (previously published in Huggins *et al*., 2019) were used as input for a Perl script to identify overlapping chromosomal regions. The script compared identified significant segments for hyperspectral traits with grain quality traits from three environments (TX08, AR09, and AR10). The identified overlapping chromosomal segments between hyperspectral traits and grain quality traits were processed with another Perl script to detect candidate genes. The above output text files, as well as the MSU7 gene annotation^32^ of the *Oryza sativa* genome was added as input for a Perl script to detect genes within a 150 kb distance to either side of the peak SNP in each region. The chromosome number, peak SNP position, gene name and distance from peak SNP were output to a text file. Additionally, Ricebase (https://ricebase.org)^39^, Oryzabase (https://shigen.nig.ac.jp/rice/oryzabase/), and SNPSeek^40^ resources were used to inspect significant segment regions and identify candidate genes.

### Statistical analysis

In this study, Vis/NIR images were analyzed to detect differences in (i) population structure, (ii) environmental differences, and (iii) quality traits. Adjusted means across years and locations of the original spectra were used in Partial Least Squares Discriminant Analysis (PLS-DA) to predict the quality variable Y matrix (group) using the process variable X matrix (Vis/NIR imaging)^41^. For the mini-core population structure study (i), the Vis/NIR hyperspectral image data of five sub-populations (P5), i.e. ‘ARO’, ‘AUS’, ‘IND’, ‘TEJ’, and ‘TRJ’, and 2 sub-species (P2), ‘AUS-IND (INDICA)’ and ‘TEJ-TRJ (JAPONICA)’ were collected and the dependent values of each category were coded as binary variables corresponding to membership (coded as 1) or non-membership (coded as zero) in the group. The ARO and AUS groups were later excluded from statistical analysis due to small sample size (4 for ARO, and 23 for AUS). Selected highly informative variables from the PLS-DA were used in a linear discriminant analysis to develop and validate a model for predicting sub-population. These variables were selected based on Variable Importance Plot (VIP) scores and coefficient values. The Vis/NIR image data for the environmental comparisons (TX08, AR09, and AR10) were also coded as binary variables corresponding to membership (coded as 1) or non-membership (coded as zero) in the group and analyzed. For both the population structure and environmental difference PLS-DA, a holdback set of 10% of the randomized samples, was used to validate the PLS-DA model developed from the training set. The training set consisted of all samples not included in the 10% holdback (validation) set. To identify specific spectral regions associated with grain traits such as AAC, ASV, bran color, grain length, width and thickness (mm) (N=197), and % grain chalk (N=137), regression analysis was performed using the imaging and grain trait data from AR09 mini-core samples. The chalk phenotype was further investigated because it showed the best correlation with imaging data. The selected range of wavelengths based on the PLS-DA model was verified by two-way clustering analysis performed by the MeV program^42^. The results were presented as a heatmap with clustering distances (Pearson’s correlation). Prior to the clustering analysis, the data for spectra and percent chalk were normalized as 0 to 1.

## Results and Discussion

### Vis/NIR phenotypic differences by sub-population

To explore whether phenotypic characteristics evaluated through a Vis/NIR hyperspectral imaging system can discern population structure, the mean percent reflectance across the spectra among the five sub-populations (abbreviated as P5) and that of the 2 sub-species (abbreviated as P2) were calculated (Supplementary Fig. S1A,B). Regression analyses of the pre-processed values were found to be similar to results using raw values since image acquisition was performed in a controlled manner, i.e. having a consistent air temperature (10 °C) and completing the imaging process within a 2-week period for all of the mini-core sets and for the bi-parental population. Therefore, these steps were omitted in further data processing. In the P5 comparisons, the reflectance of AUS was lower than the other four sub-populations in the visible (VIS) (400–700 nm) and near-infrared (NIR) (700–1000 nm) regions, however, the difference was less pronounced between 700–920 nm in the NIR region. The lower reflectance of AUS is due to a high frequency of red bran accessions in this sub-population.

PLS-DA was used to examine population structure differences within the IND, TEJ, and TRJ sub-populations using VIS (400–700 nm) and NIR ( > 700 nm) imaging data (Fig. 1). Because the red and purple grains may reduce reflectance across the spectra, accessions with these bran colors were excluded from the sub-population differentiation analysis. The sample sizes of the ARO group and the AUS group (after excluding red bran) were insufficient, therefore these groups were not tested for hyperspectral differentiation. Regression beta coefficient plots of the PLS-DA model show peaks in both positive and negative directions for classifying IND, TEJ, and TRJ groups, where peak size relates to the relationship of the wavelength predictor variable to the population classification response variable^41^, and the variable importance plot (VIP) indicates the influence of each wavelength on the predicted (sub-population) outcome (Fig. 1A). The PLS-DA actual vs predicted group classifications are shown in Fig. 1B with percent variance explained (PVE) of 29%, 62%, and 73% for TRJ, TEJ, and IND respectively. Based on coefficients and the VIP, 24 wavelengths were selected for differentiating sub-populations, as indicated by vertical lines in Fig. 1A. Of the selected wavelengths, 13 were in the visible region (400–700 nm) and 11 were in the NIR region (700–1000 nm). Within the visible spectrum, four selected wavelength peaks were found in the violet range (380–450), two in the blue range (450–495 nm), two in the green range (495–570 nm), zero in the yellow range (570–590 nm), two in the orange range (590–620 nm), and five in the red color range (620–750 nm). Selected wavelengths in portions of the NIR spectrum are in a range that is indicative of N-H stretching and C-H stretching^43^. All 24 selected wavelengths contributed to differentiating IND from TEJ and TRJ, and wavelengths at 558.6, 601.7, 659.2, 711.9, 846.0, 898.7, 917.9, and 956.2 nm contributed to differentiating TRJ from TEJ. The greater number of wavelengths differentiating IND likely is due to the greater genetic distance of IND to the TEJ and TRJ sub-populations which both belong to the Japonica subspecies. These observations were expected and are consistent with genetic differentiation between rice sub-populations and sub-species based on phenotypes, DNA markers, and sequencing^9,44^. The large oscillations in the beta coefficients between similar wavelengths, e.g. the cluster of 419.7, 434.1, and 448.5 nm and the cluster of 759.8, 769.4, and 783.8 nm, were unexpected and the reason for the pattern is unclear.

**Figure 2:**
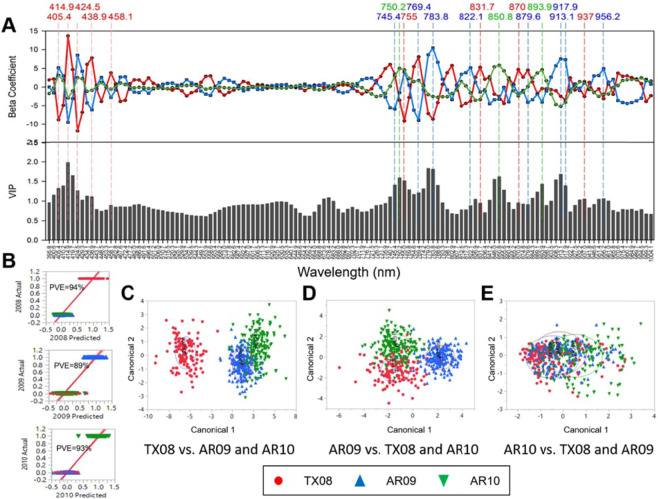
Regression beta coefficient and variable importance plot (VIP) of PLS-DA models that discriminate TX08 (red), AR09 (blue), and AR10 (green) using centered and scaled Vis/NIR spectra with the selected predictive wavelengths indicated **(A)**. Classification of TX08, AR09, and AR10 using PLS-DA models and percent variance explained (PVE) for each environment with regression line (red) **(B)**. Canonical plot for linear discriminant analysis of TX08 vs. AR09 and AR10 using the 9 predictive wavelengths selected from the PLS-DA model **(C)**, AR09 vs. TX08 and AR10 using the 8 predictive wavelengths selected from the PLS-DA model **(D)**, and AR10 vs. TX08 and AR09 using the 3 predictive wavelengths selected from the PLS-DA model **(E)**.

The 24 predictive wavelengths from the PLS-DA were used in linear discriminant analysis to develop a model for predicting sub-population from Vis/NIR data. A canonical plot from the discriminant analysis shows distinct clustering by sub-population (Fig. 1C). The discriminant analysis model achieved 70.4% prediction accuracy in the training set and 73.3% accuracy in a holdback validation set consisting of 10 randomly selected accessions from each of the three modeled sub-populations at (Supplementary Table S1). In both the holdback set, IND had the greatest prediction accuracy at 90%, followed by TRJ at 70% and TEJ at 60% correctly classified. These results indicate that genomic differences due to population structure can be detected by a combination of visible and NIR hyperspectral imaging phenotypes. The mini-core includes accessions with white, light brown, brown, red, and purple colored bran. With purple and red bran accessions excluded, we speculate that the predictive wavelengths are differentiating variation in the range of white, light brown or brown colored bran. Sub-population prediction accuracy may be increased with a larger number of accessions or greater replication per accession. Testing for hyperspectral differentiation of all five rice sub-populations would require greater representation of non-red accessions from the AUS and ARO groups.

### Vis/NIR phenotypic difference by environment

To determine whether the growing environment resulted in significant differences in grain physicochemical profiles, PLS-DA was used to examine differences between the TX08, AR09, and AR10 environments using VIS (400–700 nm) and NIR ( > 700 nm) imaging data (Fig. 2A). Because production environment does not change bran color, all grain samples were used to evaluate any environmental differences. The wavelengths differentiating among TX08, AR09 or AR10 groups were selected based on coefficient values of contrasting peaks among groups having a VIP threshold of 0.8. Wavelengths at 405.4, 414.9, 424.5, 438.9, 458.1, 755, 831.7, 870, and 937 nm contributed to differentiating TX08 from AR09 and AR10, wavelengths at 745.4, 769.4, 783.8, 879.6, 913, 917.9, and 956.2 nm contributed to differentiating AR09 from AR10 and TX08, and wavelengths at 750.2, 850.8, and 893.9 nm contributed to differentiating AR10 from AR09 and TX08. The PLS-DA actual vs predicted group classifications with PVE of 98%, 96%, and 98% for TX08, AR09, and AR10, respectively (Fig. 2B). Of the selected wavelengths, five were in the visible region (400–700 nm), and 15 were in the NIR region (700–1000 nm). Within the visible spectrum all selected wavelength peaks were found in the violet range (380–450), and discriminated TX08 from AR09 and AR10 groups. Wavelengths in the visible spectrum are known to be associated with traits such as response to biotic and abiotic stress^20,45^.

**Figure 3:**
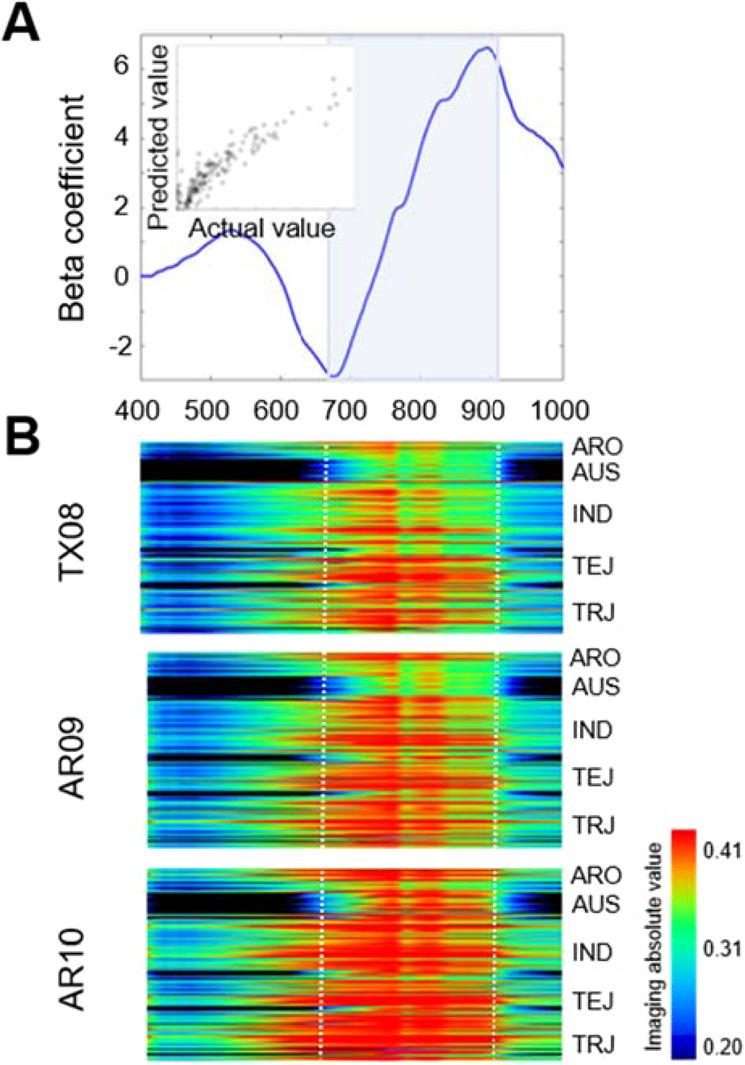
Beta coefficients of the PLS-DA model representing % chalk difference **(A)**, and a heatmap of hyperspectral relative reflectance showing absolute intensity values across populations and production environments in the Vis/NIR spectra with individuals on the y-axis. **(B)**. The x-axes for both A and B figures are wavelengths ranging from 400 to 1000 nm.

The predictive wavelengths from the PLS-DA were used in linear discriminant analysis to develop a model for predicting environmental response from Vis/NIR data. A canonical plot from the discriminant analysis shows distinct clustering of TX08 from AR09 and AR10 groups using the 9 selected predictive wavelengths (Fig. 2C), AR09 from TX08 and AR10 groups using the 8 selected predictive wavelengths (Fig. 2D), and AR10 from TX08 and AR09 groups using the 3 selected predictive wavelengths (Fig. 2E). The discriminant analysis model was used to determine % prediction accuracy in the training set and % accuracy in a holdback set consisting of 10 randomly selected accessions from each of the three modeled environments at (Supplementary Table S2, A to C). In both the training and holdback sets, TX08 vs. AR09 and AR10 comparison had the greatest prediction accuracy at 73.7%, followed by AR10 vs. TX08 and AR09 comparison at 53.9% and AR09 vs. TX08 and AR10 comparison at 32% correctly classified. These results indicate that different environmental responses can be detected by a combination of visible and NIR hyperspectral imaging phenotypes.

Soil type and weather are potential major factors that can explain observed environmental differences. In Arkansas, the soil is a Dewitt silt loam soil, whereas in Texas it is a League clay soil, and this could have produced a difference between Arkansas and Texas environments. Coupled with the weather during the rice growing season, May to November (5–7 months long because the mini-core collection contains both early and late maturing accessions) in Texas is generally warmer and extends the growing season longer than in Arkansas. The weather data for TX in 2008 and Arkansas in 2009 and 2010 indicated that the average air temperatures were higher in TX08 and AR10 compared to AR09 (Analysis of Variance, Prob> F, *p*=0.0002) and the accumulative solar radiation was higher in AR10 than in TX08 and AR09 (Analysis of Variance, Prob> F, *p* < 0.0001) during the growing season (Supplementary Fig. S3, and Table S3). The accumulative rainfall during the growing season was higher in AR09 and TX08 compared to AR10 (Analysis of Variance, Prob> F, *p*=0.012) (Supplementary Fig. S3, and Supplementary Table S3).

### Hyperspectral analysis and genotyping of grain chalk

Previous studies identified specific NIR spectral regions detecting starch, protein, and fat content, and weight of grains^14–16^, demonstrating hyperspectral imaging systems as a potential means of detecting grain quality traits. We questioned whether variation in various rice grain quality traits in the mini-core can be detected using the hyperspectral imaging system. The chalk phenotype became the primary focus among grain traits we tested, because: 1) in rice it is a very important grain trait that affects crop value ^1,2^, 2) it showed the most significant correlation with hyperspectral data, and 3) a bi-parental mapping population segregating for percent chalk that was previously phenotyped^24^ allowed us to verify specific spectral regions identified from the USDA mini-core collection GWAS.

To identify Vis/NIR spectral regions that are associated with grain quality traits, we first performed K-means clustering analysis of hyperspectral imaging data from a single environment (AR09 as the representative environment). The Vis/NIR spectral regions were divided into several groups based on clustering of wavelengths. This resulted in the identification of five distinctive wavelength groups (Groups 1 to 5), which included spectral regions of 395–424, 429–587, 592–702, 702–922, and 927–1004 nm, respectively (data not shown).

PCA was performed using the hyperspectral image data for Groups 1 to 5, and the resulting first principal component (PC1) (accounting for> 95% of the variation) values were regressed with actual grain quality trait values. Significant correlations (*p* < 0.05) were found with percent chalk, percent amylose, ASV, and kernel bran color (white, light brown, and brown classes only), width, length, and thickness traits, with R^2^ values ranging from 0.09 to 0.89. The highest R^2^ was found between the chalk phenotype and the PC1 for hyperspectral data in the 702–922 nm region (Group 4) (R^2^ = 0.93, *p* < 0.0001) (Table 1). To further evaluate the 702–922 nm region that is closely associated with percent chalk, a PLS-DA model was developed, and it was determined that wavelengths in the range of 690–920 nm were highly correlated with percent chalk (Fig. 3A). This region was also consistent across production environments, i.e. TX08, AR09, and AR10 (Fig. 3B) and encompassed the NIR regions that distinguished the three environments (Fig. 2A).

**Table 1:** ANOVA result of five Vis/NIR spectral groups displaying phenotypic variation for grain traits, % chalk, % amylose, ASV, bran color, width, length, and thickness among white grain samples from the AR09 environment. R^2^ values are presented between PC1 components per wavelength group and grain quality trait.

	Group 1		Group 2		Group 3		Group 4		Group 5	
	R^2^	Prob > F	R^2^	Prob > F	R^2^	Prob > F	R^2^	Prob > F	R^2^	Prob > F
% Chalk	0.81	**	0.74	**	0.83	**	0.89	**	0.82	**
Amylose %	0.31	**	0.31	**	0.36	**	0.31	**	0.31	**
ASV	0.02	ns	0.02	ns	0.02	ns	0.01	ns	0.01	ns
Kernel Bran Color	0.29	**	0.36	**	0.26	**	0.16	**	0.28	**
Kernel Width mm	0.21	**	0.20	**	0.23	**	0.19	**	0.20	**
Kernel Length mm	0.01	ns	0.00	ns	0.01	ns	0.02	ns	0.01	ns
Kernel Thickness (mm)	0.11	**	0.10	ns	0.14	**	0.11	**	0.10	ns

**, * and ns indicate p < 0.01, p < 0.05 and p > 0.05, respectively.

**Figure 4:**
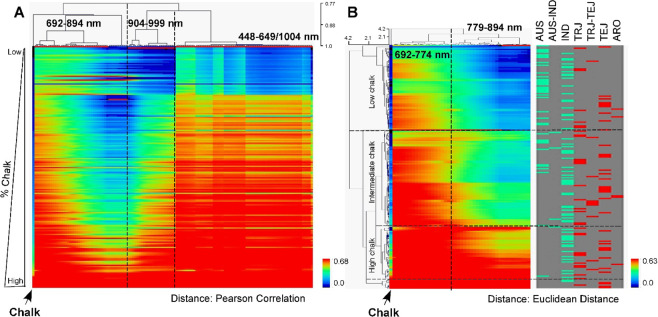
Hierarchical clustering heatmap of the hyperspectral images for AR09 samples and their % chalk in Vis/NIR spectra **(A)** and the selected region (692–894 nm) associated with % chalk clustered by Euclidean distance of individuals on the x-axis and wavelengths on the y-axis with subpopulation assignments of individual lines indicated on the far right **(B)**. r.u. stands for relative unit.

One-way hierarchical clustering analysis using AR09 samples further verified spectral regions which showed a similar pattern of percent chalk in the mini-core (Fig. 4A). Three distinct clusters at 448–649 nm, 1004 nm, 692–894 nm, and 904–999 nm were identified. Among those clusters, the 692–894 nm region was most strongly associated with percent chalk phenotype (Fig. 4A). Region 692–894 nm was further studied to determine more specifically which wavelengths were associated with percent chalk when categorized as high, intermediate, or low chalk using the Euclidean distance metric. Hierarchical cluster results showed that region 692–774 nm better separated low chalk accessions from intermediate and high chalk accessions, while the 779–894 nm region distinctly separated low from intermediate, and intermediate from high chalk accessions (Fig. 4B). There was no obvious relationship between sub-species and percent chalk categories (Fig. 4B).

**Figure 5:**
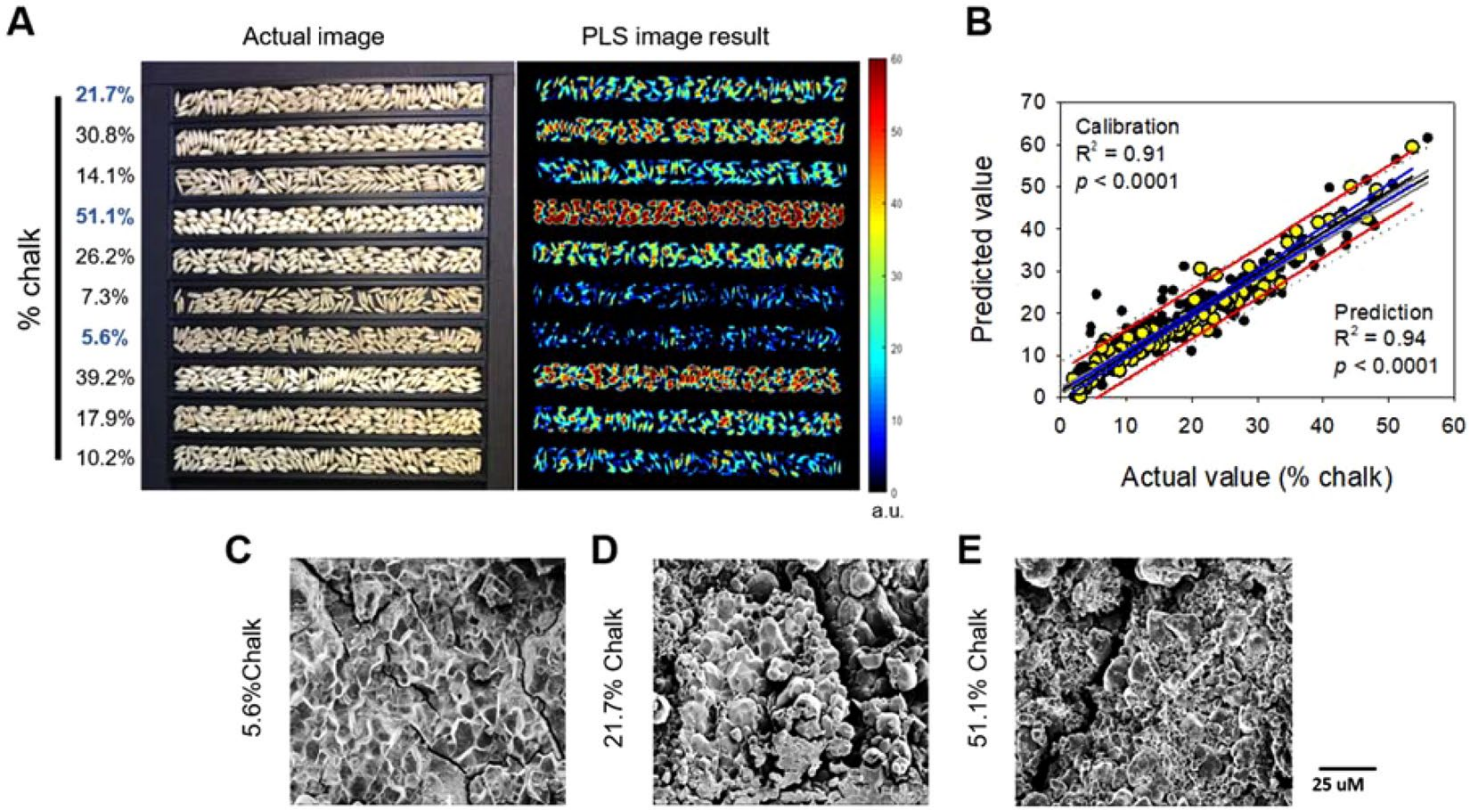
PLS image of a subset of KZ-RILs with diverse levels of grain chalk **(A)**, linear regression of predicted values of a PLS-DA model using the 702–922 nm range as a function of actual % chalk observed in brown rice of the KZ-RIL population **(B)**, and scanning electron microscope (SEM) images of low **(C)**, intermediate **(D)**, and high chalk KZ-RILs **(E)**. a.u. in (A) stands for arbitrary unit. Black and yellow dots in (B) display calibrated and predicted values, respectively. Grey dotted line/red solid line, grey solid line/blue solid line, and black lines in (B) represent 95% prediction and confidence bands and regression of calibrated/predicted values, respectively.

Diversity panels can be an excellent genetic resource to identify chromosomal regions associated with various phenotypic traits. However, diverse genetic backgrounds can have confounding factors that may mask or produce false associations with the phenotype of interest. Therefore, we used the bi-parental recombinant inbred line population, KBNT-lpa1 x ZHE733 (KZ-RIL), that is segregating for percent chalk to verify that the 702–922 nm region (from Fig. 4A) can differentiate chalk phenotypes from a common genetic background. Hyperspectral images of the grain samples of the KZ-RIL population grown in two different years were captured (Fig. 5A, selected extremes), and the 702–922 nm region was used to develop a PLS-DA model. Based on this model, the calibrated correlation coefficient was 0.91 and the predicted correlation coefficient was 0.94 (Fig. 5B). Among the selected KZ-RILs having divergent percent chalk (Fig. 5A), SEM images showed that the RIL with high chalk (51.1%) had an overall disorganized and irregular packing of starch granules (Fig. 5E). Conversely, the RIL with low chalk (5.6%) had a regular and organized cellular structure (Fig. 5C), and the RIL with intermediate chalk (21.7%) had a cellular structure that was less organized than the RIL with high chalk and more irregular than the RIL with low chalk (Fig. 5D). Notably, the high chalk RIL had more air spaces between starch granules than the low chalk RIL as has been reported previously^46^.

**Figure 6:**
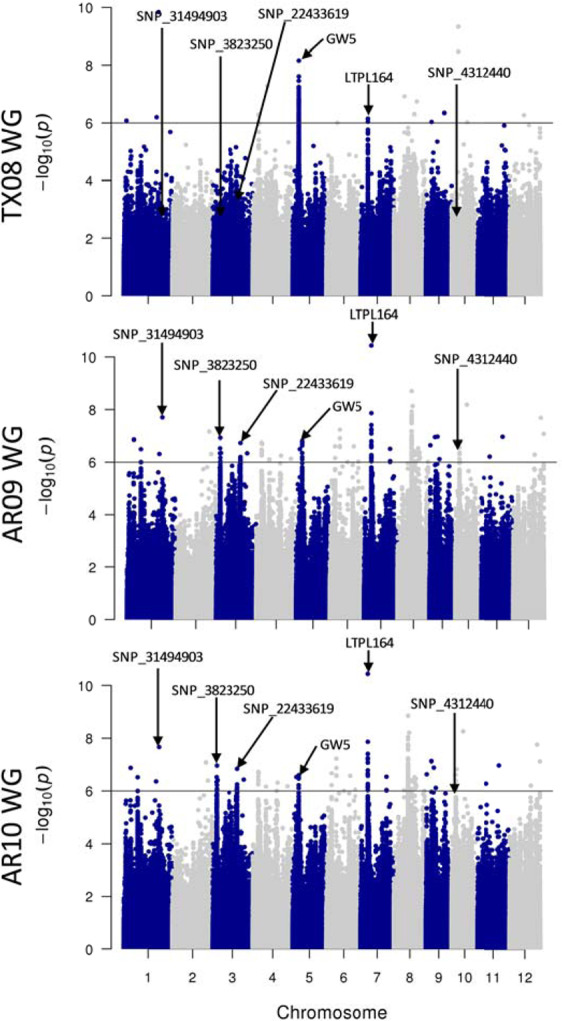
Manhattan plots for GWAS of the first principal component for the hyperspectral region 707–922 nm for environments TX08, AR09 and AR10. Only non-pigmented bran accessions are presented in this analysis. The x-axis displays chromosome pseudomolecule coordinates and the y-axis displays the –log_10_(p) value for each SNP across chromosomes. The dark horizontal line represents the genome-wide significance threshold. The labeled SNPs are discussed as potential candidates in the text. GW5 – grain weight 5 gene; LTPL164 – Protease inhibitor/seed storage gene.

PC1 calculated from the 702–922 nm wavelengths of the hyperspectral image data collected from the mini-core accessions was used for GWAS analysis. Because bran color can mask chalk and affect wavelength absorption, reflectance and transmittance (Figure S3), the analysis of only non-pigmented bran accessions was performed to reduce the possibility of confounding effects and false associations. A total of 44 chromosomal candidate regions were identified as associated with spectral PC1 values across two or more environments (Fig. 6; Supplementary File S1). Seventeen segments associated with the first principal component of the hyperspectral values (hyperspectral PC1) were identified for TX08, 49 for AR09, and 50 for AR10. Forty-eight segments were common between AR09 and AR10. Five hyperspectral PC1-associated segments were common between TX08, AR09, and AR10 including two of the segments located on chromosome 4, one on chromosome 5, one on chromosome 7, and the other on chromosome 9 (Supplementary File S1). The 68 non-overlapping chromosomal segments from all three environments associated with hyperspectral PC1 were examined for overlap with percent chalk, AAC, ASV, and grain length and width segments detected by GWAS in the mini-core by Huggins *et al*., (2019) to identify shared chromosomal segments. Grain chalk-associated segments overlapped with other hyperspectral grain trait-associated segments from two or more environments for 21 different loci. The hyperspectral-chalk associated segments also overlapped with 13 AAC-associated segments, one ASV segment, and two grain dimension segments previously reported by Huggins *et al*., (2019). Some of the significant hyperspectral chalk segments contained characterized known genes or were proximal to them. On chromosome 3, a segment was proximal to the grain size/plant height TIFY11b gene and another co-located with a phosphatidylinositol-4-phosphate 5-kinase gene (PIPK) for AR09 and AR10 (Fig. 6; Supplementary File S1). A segment identified on chromosome 5 for AR09 and AR10 co-located with the characterized chalk5 gene, an inorganic H + pyrophosphatase. Another segment on chromosome 5, detected in TX08, AR09, and AR10, was proximal to the characterized grain dimension gene, Grain Weight 5 (DQ991205). A segment on chromosome 7, also detected in the three environments, co-located with a seed storage/protease inhibitor gene (LTPL164) (Fig. 6; Supplementary File S1).

The shared significant chromosomal segments detected between spectral regions associated with chalk and other grain quality traits were used to identify associated candidate genes. Gene annotations, biological and molecular functions were used to propose candidate rice genes that fell within 150 kb on either side of peak SNP in significant segments. We identified 12 potential candidate genes related to starch and sucrose that met the criteria and overlapped with grain chalk. Several of these candidate genes have vital roles in starch or sucrose biosynthesis. The candidate *LOC_*Os01g54560, is a trehalose phosphate synthase gene on chromosome 1, ~117 kb upstream of SNP-31,494,903 (the number included in the SNP name represents the physical position in base pairs of the SNP on the MSU7 pseudomolecule assembly) (Table 2). The synthesis of trehalose can occur via multiple pathways, but the best known involves trehalose-6-phosphatase, which is a known regulator of plant sucrose^47,48^. Trehalose synthesis and accumulation in plant tissues induce sucrose synthase activity^49^, thus affecting sucrose and starch biosynthetic activity. Notably, trehalose accumulation in varying plant tissues can be triggered by abiotic stresses such as oxidation, heat, and drought^50^, where they mitigate the stress effects. The candidate *LOC_Os03g07480*, is a sucrose transporter (SUT1) gene, ~19 kb upstream of SNP-3,823,250 on chromosome 3 (Table 2). Sucrose transporters act as distribution centers for photo-assimilates and move sucrose into the phloem of most plants by active transport^51–53^. However, in rice, the SUT1 not only functions in phloem loading but may be involved in sucrose transportation to the sink (grain)^53–56^. The SUT1 gene is usually expressed in panicles, leaf sheaths, and leaf blades after heading, and promotes the mobilization of starch to sink tissue^57–59^, thus playing a key role in grain filling and quality. Candidate *LOC_Os03g40270*, ~49 kb upstream of SNP-22,433,619, is an α-1,4-glucan protein synthase (α-1,4-glucanotransferase) gene located on chromosome 3 (Table 2). The α-1,4-glucan protein synthase gene belongs to a class of enzymes called disproportionating enzymes (DPE)^60–62^. Previous studies have linked this enzyme with starch granule biosynthesis, more specifically, amylopectin structure and a role in starch degradation^62–64^. When this disproportionating enzyme was suppressed, it resulted in reduced amylopectin chains and increased amylose, leading to loosely packed starch granules in the endosperm^65^. Hence, α-1,4-glucan protein synthase contributes to the complex processes regulating grain filling. The candidate *LOC_Os10g08022*¸ is characterized as a fructose-bisphosphate aldose (FBA) gene, ~31 kb downstream of SNP-4,312,440 on chromosome 10 (Table 2). FBAs catalyze D-glyceraldehyde-3-phosphate (GAP) and dihydroxyacetone phosphate (DHAP) from D-fructose-1,6-bisphosphate (FBP) and is vital to glycolysis and gluconeogenesis^66–68^. FBAs also play a key role in regulating both starch and sucrose biosynthesis in plants^69–71^. Two forms of FBA exist, cytosolic FBA which is involved in sucrose synthesis^71,72^, and chloroplastic FBA which is vital in starch biosynthesis^69^. Moreover, inhibition of cytosolic FBA led to decreased sucrose biosynthesis but increased starch biosynthesis^72,73^. Notably, FBAs not only regulate starch and sucrose biosynthesis but play pivotal roles in various biological, metabolic and physiological pathways that require sucrose, including response to abiotic stresses^71,74–76^.

**Table 2:** Summary of common candidate loci between hyperspectral results and chalk trait in the mini-core diversity panel.

Chr	Peak SNP (bp)	P	R^2^	Candidate gene	Description
1	28,782,853	4.86 × 10^−7^	0.24	LOC_Os01g50060	1-aminocyclopropane-1-carboxylate deaminase
1	31,494,903	1.99 × 10^−8^	0.26	LOC_Os01g54560 ^¤ ‡^	Trehalose synthase
3	3,823,250	1.20 × 10^−7^	0.25	LOC_Os03g07480 ^¤ ‡^	Sucrose transporter (SUT1)
3	22,433,619	1.84 × 10^−8^	0.27	LOC_Os03g40270 ^¤ ‡^	Alpha-1,4-glucan-protein synthase
3	28,376,883	4.58 × 10^−7^	0.27	LOC_Os03g49800	Phosphatidylinositol-4-phosphate 5-kinase (PIPK)
4	21,277,848	7.00 × 10^−7^	0.18	LOC_Os04g35030	Cellulose synthase-like protein (CSLH3)
4	22,079,754	4.81 × 10^−7^	0.24	LOC_Os04g36610 ^¤^	Endoglucanase
5	3,349,202	3.16 × 10^−7^	0.29	LOC_Os05g06480	Inorganic H + pyrophosphatase (chalk5)
6	4,719,289	9.52 × 10^−8^	0.26	LOC_Os06g09450 ^¤^	Sucrose synthase (SUS2)
8	15,723,764	6.76 × 10^−8^	0.26	LOC_Os08g25734	Glucose-1-phosphate adenylyltransferase (AGPS2)
10	4,312,440	2.16 × 10^−7^	0.28	LOC_Os10g08022 ^¤ ‡^	Fructose-bisphosphate aldolase isozyme

^‡^ - denotes candidate loci discussed in discussion.

^¤^ - denotes candidate loci associated with grain chalk segments.

The results of this study demonstrate that hyperspectral imaging offers a high-throughput, efficient method of assessing rice grain traits that otherwise require labor and time-consuming assays. With this method, large amounts of data are captured, and multiple traits can be characterized simultaneously. Hyperspectral imaging technique provides a quantitative assessment of grain quality that is repeatable and not dependent on subjective ratings (e.g., ASV). NIR hyperspectral imagery consists of numerous bands with small spectrum gaps (every 4 nm in our study) and can assess grain traits such as fat, starch, protein, moisture, color, and many other physicochemical compounds at once. The involvement of multiple bands in the prediction of chalk suggests that there may be multiple mechanisms leading to chalky grains that each have different impacts on the hyperspectral profile. The spectral regions associated with chalk could be further refined for use in a multispectral apparatus for high-throughput quantification of chalk in rice grains. Such an apparatus may improve the efficiency of selection for grain quality in rice breeding programs.

GWAS was used to confirm known genes and to identify novel candidate genes affecting grain quality traits using hyperspectral imaging. The PLS-DA models of hyperspectral data identify spectral ranges that distinguish genetic and production environment differences, and this information may help to resolve the genetics of complex traits such as rice grain quality.

## Supplementary information

Supplementary Figures

Supplementary Tables

Supplementary File S1.
