# Supplementary material for: Vis/NIR hyperspectral imaging distinguishes sub-population, production environment, and physicochemical grain properties in rice
Source: Sci Rep. 2020 Jun 9;10:9284. doi: 10.1038/s41598-020-65999-7 (PMC7283329; doi:10.1038/s41598-020-65999-7)
Supplement: Supplementary file 1 — Supplementary Figures [file 41598_2020_65999_MOESM1_ESM.docx]

**Supplementary Figure S1**. Mean ± standard error (SE) percent reflectance of spectra for P5 **(A)** and P2 **(B)**. Purple line, ARO; pink line, AUS; red line, IND; blue line, TEJ; green line, TRJ in the P5 (A), and red line, AUS-IND; blue line, TEJ-TRJ in the P2 (B).


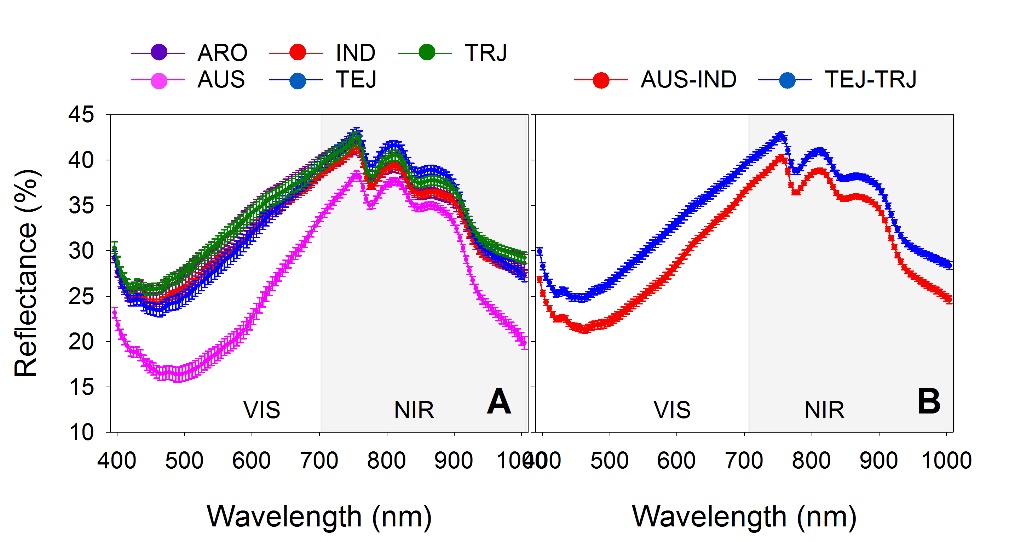


**Supplementary Figure S2**. Mean ± standard error (SE) percent reflectance of spectra of all grain samples for three environments AR09 (blue), AR10 (red), and TX08 (green).


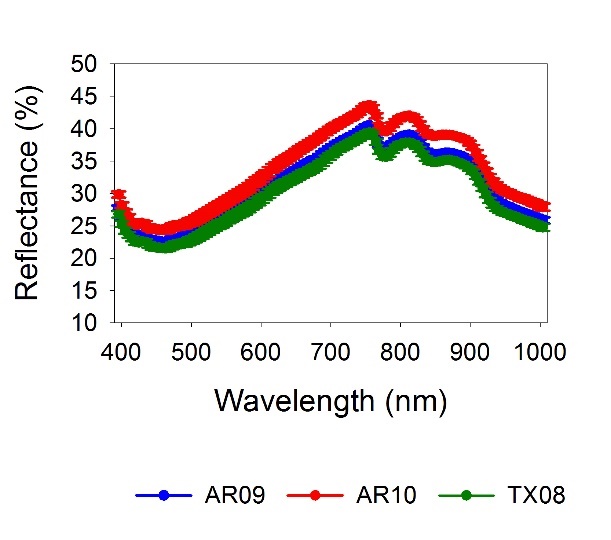


**Supplementary Figure S3.** Average daily temperature **(A)** and total daily solar radiation **(B)** during rice growing season, May to November in Texas 2008 (blue), Arkansas 2009 (orange), and Arkansas 2010 (grey).

**A**

**B**

**Supplementary Figure S4.** Q-Q plots for GWAS of the first principal component for the hyperspectral region 707 – 922 nm for environments TX08, AR09 and AR10 in the mini-core diversity panel. The plots are for the analysis of non-pigmented bran only. The quantile-quantile plot x-axis displays the expected distribution of association for SNPs and the y-axis display the observed SNP distribution in –log10(p).

**
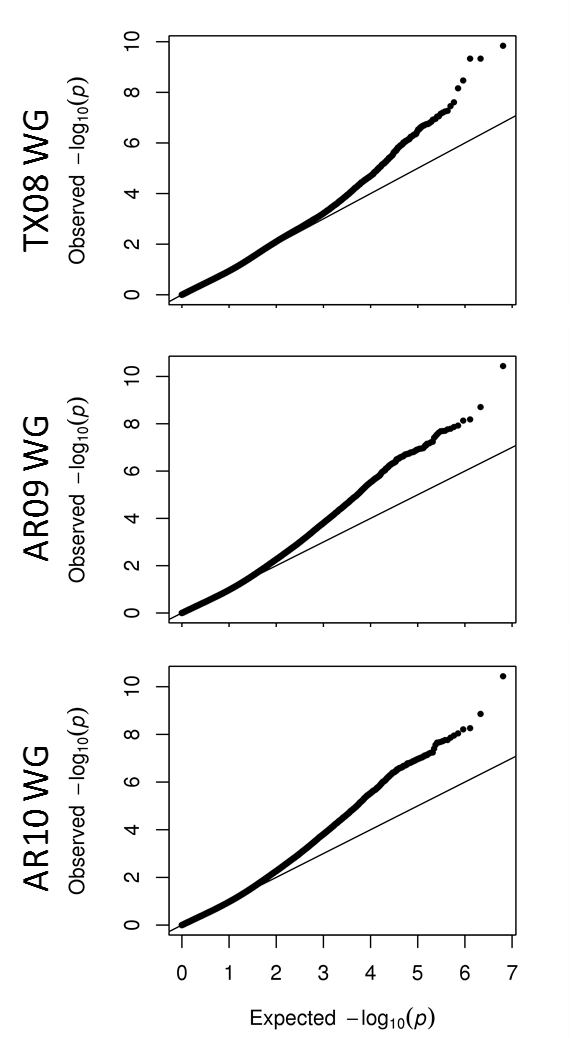
**
