# Supplementary material for: Vis/NIR hyperspectral imaging distinguishes sub-population, production environment, and physicochemical grain properties in rice
Source: Sci Rep. 2020 Jun 9;10:9284. doi: 10.1038/s41598-020-65999-7 (PMC7283329; doi:10.1038/s41598-020-65999-7)
Supplement: Supplementary file 2 — Supplementary Tables [file 41598_2020_65999_MOESM2_ESM.docx]

**Supplementary Table S1.** Confusion matrix (by percent) of linear discriminant analysis of TRJ, TEJ, and IND subpopulations using the 24 predictive wavelengths selected from the PLS-DA model of Vis/NIR (400-1000 nm). The discriminant model was validated using a random holdback set per subpopulation.

| Vis-NIR | Actual | % predicted | | |
| --- | --- | --- | --- | --- |
|  | Subpop | IND | TEJ | TRJ |
| Training | IND | 67 | 13 | 20 |
|  | TEJ | 22 | 72 | 6 |
|  | TRJ | 20 | 4 | 76 |
| Holdback | IND | 90 | 0 | 10 |
|  | TEJ | 20 | 70 | 10 |
|  | TRJ | 30 | 10 | 60 |

**Supplementary Table S2A.** Confusion matrix (by percent) of linear discriminant analysis of TX08 vs. AR09 and AR10 using the 9 predictive wavelengths selected from the PLS-DA model of Vis/NIR (400-1000 nm). The discriminant model was validated using a random holdback set per subpopulation.

| Vis-NIR | Actual | % predicted | | |
| --- | --- | --- | --- | --- |
|  | Environment | AR09 | AR10 | TX08 |
| Training | AR09 | 52.1 | 29.9 | 18.0 |
|  | AR10 | 33.9 | 55 | 11.1 |
|  | TX08 | 16.8 | 9.5 | 73.7 |
| Holdback | AR09 | 34.6 | 29.6 | 35.8 |
|  | AR10 | 25.1 | 55.7 | 19.2 |
|  | TX08 | 16.7 | 15.8 | 67.5 |

**Supplementary Table S2B.** Confusion matrix (by percent) of linear discriminant analysis of AR09 vs. TX08 and AR10 using the 8 predictive wavelengths selected from the PLS-DA model of Vis/NIR (400-1000 nm). The discriminant model was validated using a random holdback set per subpopulation.

| Vis-NIR | Actual | % predicted | | |
| --- | --- | --- | --- | --- |
|  | Environment | AR09 | AR10 | TX08 |
| Training | AR09 | 32.0 | 28.9 | 39.2 |
|  | AR10 | 19.4 | 53.9 | 26.7 |
|  | TX08 | 12.4 | 21.2 | 66.4 |
| Holdback | AR09 | 34.1 | 27.3 | 38.6 |
|  | AR10 | 18.6 | 55.9 | 25.5 |
|  | TX08 | 12.2 | 20.3 | 67.5 |

**Supplementary Table S2C.** Confusion matrix (by percent) of linear discriminant analysis of AR10 vs. TX08 and AR09 using the 3 predictive wavelengths selected from the PLS-DA model of Vis/NIR (400-1000 nm). The discriminant model was validated using a random holdback set per subpopulation.

| Vis-NIR | Actual | % predicted | | |
| --- | --- | --- | --- | --- |
|  | Environment | AR09 | AR10 | TX08 |
| Training | AR09 | 21.6 | 30.9 | 47.4 |
|  | AR10 | 18.9 | 57.2 | 23.9 |
|  | TX08 | 10.2 | 22.6 | 67.2 |
| Holdback | AR09 | 22.3 | 28.5 | 49.2 |
|  | AR10 | 15.6 | 58.8 | 25.6 |
|  | TX08 | 9.9 | 24 | 66.1 |

**Supplementary Table S3.** Monthly air temperature, solar radiation, and rainfall as well as pairwise comparisons using Tukey HSD at the USDA research station during rice growing season, May to November in Texas 2008 (TX08), Arkansas 2009 (AR09), and Arkansas 2010 (AR10).

|  | **Avr monthly temp (°C)** | | | | | | **Sum monthly solar radiation** | | | | | | **Sum monthly rainfall (in)** | | | | | |
| --- | --- | --- | --- | --- | --- | --- | --- | --- | --- | --- | --- | --- | --- | --- | --- | --- | --- | --- |
|  | **2008 TX** | | **2009 AR** | | **2010 AR** | | **2008 TX** | | **2009 AR** | | **2010 AR** | | **2008 TX** | | **2009 AR** | | **2010 AR** | |
|  | **LS mean** | **HSD** | **LS mean** | **HSD** | **LS mean** | **HSD** | **LS mean** | **HSD** | **LS mean** | **HSD** | **LS mean** | **HSD** | **LS mean** | **HSD** | **LS mean** | **HSD** | **LS mean** | **HSD** |
| **May** | 24.37 | A | 20.95 | B | 23.12 | A | 20.99 | B | 18.82 | B | 25.77 | A | 0.13 | A | 0.32 | A | 0.12 | A |
| **Jun** | 27.06 | A | 27.00 | A | 28.22 | A | 21.47 | B | 24.83 | A | 27.60 | A | 0.24 | A | 0.10 | AB | 0.03 | B |
| **July** | 27.58 | A | 25.28 | B | 27.58 | A | 22.38 | B | 21.30 | B | 27.68 | A | 0.19 | A | 0.32 | A | 0.05 | A |
| **Aug** | 27.16 | A | 24.98 | B | 28.41 | A | 19.45 | B | 23.55 | A | 24.85 | A | 0.37 | A | 0.15 | A | 0.12 | A |
| **Sep** | 24.56 | A | 22.56 | B | 24.29 | A | 17.11 | B | 15.91 | B | 21.78 | A | 0.29 | A | 0.13 | A | 0.02 | A |
| **Oct** | 17.10 | AB | 14.80 | B | 17.37 | A | 15.07 | A | 9.51 | B | 18.02 | A | 0.06 | B | 0.44 | A | 0.02 | B |
| **Nov** | 15.85 | A | 12.31 | B | 11.56 | B | 13.00 | A | 11.14 | A | 10.92 | A | 0.16 | A | 0.08 | A | 0.22 | A |
| **Accumulative amounts during growing season** | | | | | | | 3962.30 | **B** | 3824.65 | **B** | 4794.62 | **A** | 43.97 | **A** | 47.20 | **A** | 17.50 | **B** |
